# The Effect of the Transition to Home Monitoring for the Diagnosis of OSAS on Test Availability, Waiting Time, Patients' Satisfaction, and Outcome in a Large Health Provider System

**DOI:** 10.1155/2014/418246

**Published:** 2014-04-24

**Authors:** Ahmad Safadi, Tamar Etzioni, Dan Fliss, Giora Pillar, Chen Shapira

**Affiliations:** ^1^Department of Otolaryngology Head and Neck and Maxillofacial Surgery, Tel Aviv Sourasky Medical Center, 64239 Tel Aviv, Israel; ^2^Sleep Clinic, Clalit Health Services, Carmel Medical Center, Technion Faculty of Medicine, 34362 Haifa District, Israel; ^3^Lady Davis Carmel Medical Center, Technion Faculty of Medicine, 34362 Haifa, Israel

## Abstract

During 2009, the Haifa district of Clalit Health Services (CHS) has switched from in-lab polysomnography (PSG) to home studies for the diagnosis of obstructive sleep apnea (OSA). We assessed the effects of this change on accessibility, waiting time, satisfaction, costs, and CPAP purchase by the patients. Data regarding sleep studies, CPAP purchase, and waiting times were collected retrospectively from the computerized database of CHS. Patients' satisfaction was assessed utilizing a telephone questionnaire introduced to a randomized small sample of 70 patients. Comparisons were made between 2007 and 2008 (in-lab PSGs) and 2010 and 2011 (when most studies were ambulatory). Of about 650000 insured individuals in the Haifa district of CHS, 1471 sleep studies were performed during 2007-2008 compared to 2794 tests during 2010-2011. The average waiting time was 9.9 weeks in 2007-2008 compared to 1.1 weeks in 2010-2011 (*P* < 0.05). 597 CPAPs were purchased in 2007-2008 compared to 831 in 2010-2011. The overall patients' satisfaction was similar, but discomfort tended to be higher in the in-laboratory group (4.1 vs 2.7 in a scale of 0–10; *P* = 0.11). Switching to ambulatory diagnosis improved the test accessibility and reduced the waiting times. Patients' satisfaction remained similarly high. The total direct cost of OSA management was reduced.

## 1. Introduction 

Obstructive sleep apnea syndrome (OSAS) affects about 5% of the adult population older than 50 years. It has been shown that OSAS is strongly related to cardiovascular morbidity and mortality [1], hypertension [2, 3], neurocognitive development and behavioral disturbances [4], and road accidents [5]. OSAS has an important effect on quality of life. The most prevalent symptoms include snoring, day-time sleepiness, fatigue, memory and mood disturbances, and impotence [6, 7]. Apparently, it is of utmost importance to diagnose and treat people with OSA syndrome. The gold standard for the diagnosis of OSAS is still considered a full night in the lab polysomnography (PSG), but it is time consuming and expensive, making a long waiting list. Additionally, PSG requires an overnight stay in the lab which makes it uncomfortable for many patients. In order to facilitate the diagnostic process, many portable monitoring devices have been suggested for home sleep studies ranging from a single channel device (pulse oximetry) to full in-home PSG [8–30]. In 2007, a task force of the American Academy of Sleep Medicine (AASM) recommended the use of portable monitoring devices for population with high risk for moderate to severe OSAS and without comorbidities [8], although they recommended that a negative test should lead to PSG. For this recommendation, the AASM proposed the use of a type 3 portable monitoring device which includes at least 4 channels, including airflow, respiratory effort measurements, and pulse oximetry recordings [9]. Several studies which compared portable devices to PSG found similar results in terms of apnea-hypopnea indices (AHI's), usually with favorable costs [10–12, 14, 24–26]. Still, most of the recorders record respiratory channels but not sleep and thus the denominator for the calculation of respiratory indices is recording time rather than sleep time. Based on the known association between sympathetic activation and sleep apnea [31], a novel device based on autonomic activation recording has been developed [13–21]. This device is based on an actigraph, peripheral arterial tone (PAT) recording, pulse rate, and pulse oximetry. The Watch-Pat 100 has been shown to provide a reasonably accurate assessment of the AHI compared to PSG [13–17] and is widely used in Clalit Health Services (CHS) in Israel.

Clalit Health Services (CHS) is the largest health insurance provider in Israel, insuring more than 4 million people. CHS uses patient electronic medical record for more than 25 years. The database is much well kept and several previous studies have utilized partial data of this registry in the field of sleep [32–34]. There is a very prompt database management in this closed population, providing accurate and representative information. Haifa and West Galilee district of this organization is a semiautonomic subgroup of it, insuring about 650000 people, having its' own sleep clinic and sleep lab system. Many of the insured patients in these areas live in relative rural areas and are in low socioeconomic status; thus compliance to medical examinations that need long travels are low. During 2009, Haifa and West Galilee district had made a decision to switch from in-lab diagnosis to diagnosis in the patients' home. 2009 was a transition year and thereafter (2010 and on) over 70% of the sleep studies due to suspected OSA were performed in home. In the current study we assessed the effects of this change on accessibility, waiting time, satisfaction, costs, and short term outcome (CPAP purchase) of the patients.

## 2. Methods

The study has a retrospective component of retrieving data from the CHS database, as well as a small prospective sample of satisfaction questionnaire administration. Both parts have been approved by the Carmel (the hospital of Haifa district of CHS) hospital's institutional review board (IRB).

### 2.1. The Retrospective Study Design

The database inquiry consisted of all adult population (aged 18 and above) of Haifa district of CHS. Data regarding the amount of sleep studies, amount of acquired CPAPs, and waiting times between request and study performance were collected retrospectively from the computerized database and were compared between the years 2007 and 2008 representing the in-laboratory studies and 2010 and 2011 when most of the studies were ambulatory (76% of the tests). Since the inflation rate in Israel during these years was relatively very low, the cost was evaluated in the local coin (shekels) without adjustment to inflation rates.

### 2.2. The Prospective Study Design

Patients' satisfaction was tested by a specific satisfaction questionnaire which included six items and was filled via a telephone call with the researcher (AS). For this purpose, a small sample of patients from each time was selected. 25 patients from each period (2007-2008 and 2010-2011) were randomly selected from the patients' lists for those years, and they were contacted by phone. In order to minimize a memory recall bias, additional 20 patients who underwent in-laboratory full night PSG during 2011 were also contacted by phone and completed the same questionnaire. These results were compared primarily not only to the 2007-2008 data to learn n potential memory recall bias, but also to 2010-2011 to compare convenience.

### 2.3. Statistical Analysis

We compared the data for 2007-2008 with that of 2010-2011 using *t*-test and chi-square test for proportions. *P* < 0.05 was considered statistically significant.

## 3. Results

**Figure 1:**
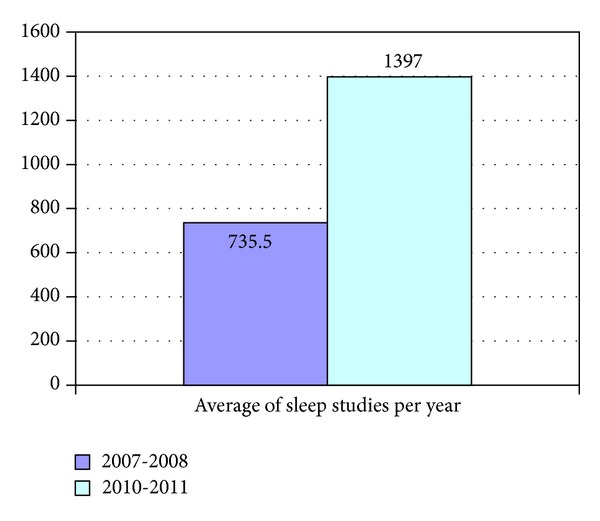
The average of sleep studies performed per year during 2007-2008 compared to 2010-2011.

Since 2009 was a transition year, the data from that year was not included in the current study. Excluding 2009, between January 2007 and December 2011, 4265 sleep studies were performed at CHS of Haifa district. There was a significant increase in the amount of sleep studies performed upon these years. While 1471 sleep studies were done during 2007-2008 (735.5 studies/year), 2794 sleep studies (1397 studies/year) were done during 2010-2011 (*P* < 0.05) (Figure 1). This represents a 90% increase in the number of sleep studies performed, while the change in the number of insured people between these years was less than 5%.

The average waiting time to a sleep study (defined as the time between request and performance of a sleep study as appears in the database) decreased significantly, from 9.9 weeks during 2007-2008 to 1.1 weeks during 2010-2011 (*P* < 0.005). One interesting finding was the trend toward shortening of the waiting time for an in-lab PSG in 2010-2011 compared to that in 2007-2008 (4.8 weeks versus 9.9 weeks; *P* = 0.19).

The amount of CPAPs purchased during 2007-2008 was 597 devices, compared to 831 devices during 2010-2011. This increase is small (39%) compared to the almost doubling (90% increase) of the amount of sleep studies performed during these years.

Twenty-one patients who underwent PSG during 2007-2008 (of 25 who were approached, 84% response rate), 18 patients who underwent PSG during 2011 (of 20 who were approached, 90% response rate), and 25 patients who underwent home study during 2010-2011 (100% response rate) filled the satisfaction questionnaires. The questionnaire analyses results of patients studied by PSG were not significantly different between those studied in 2007-2008 and those in 2011, which indicates a relatively low possibility of recall bias. In order to reduce this bias effect in the analyses, data from patients who underwent PSG during 2011 was combined to data of patients who underwent PSG during 2007-2008 (overall 39 patients). Results of patients' satisfaction are summarized in Table 1.

Despite the significant increase in the amount of sleep studies upon these years, the total direct expense (i.e., cost of sleep studies, without counting for indirect reduced expenses (health care utilization) due to increased rate of treated patients) decreased. The cost of a home study was less than one-third of the cost of an in-lab PSG. Therefore, despite the 90% increase in the amount of sleep studies, the shift to home studies was accompanied by an over 20% decrease in the total expense on diagnosis of OSA. In 2007-2008 1471 PSGs were performed with a total cost of approximately $500,000 (average of $340 per study, with a range of $300–$350 depending on sort of study, capping, and lab). The total cost of diagnostic studies in 2010-2011 was $398,000. This consisted of 660 PSGs with a total cost of $195,000 (average of almost $300 per PSG) + 2134 home studies with a total cost of $203,000 (average of almost $100 per ambulatory study).

## 4. Discussion

We found a significant increase in the amount of sleep tests performed during 2010-2011 compared to that during 2007-2008, which was accompanied by a significant shortening of waiting times and cost. This finding represents the advantages of home sleep testing: better accessibility and reduced cost. The increase in total amount of studies indicates that either prior to this change sleep studies were under performed or there was an increase in demand induced by supply. Either way, the transition from lab to home studies resulted in increased number of studies, yet waiting time was reduced, cost was reduced, and satisfaction remained high. In the USA, it is estimated that 80% of patients are undiagnosed [35]. This was explained by the limited availability of sleep laboratories; while the request of sleep studies was increased by 12-fold in the last decade, the number of sleep laboratories was only doubled [36].

Considering all these years together, our data indicate that 178 sleep tests were done annually per 100,000 people, with a waiting time of 1.1–9.9 weeks. This finding represents an overall reasonable level of accessibility to sleep evaluation in our region as compared to the literature reports. For example, in the UK, the number of sleep tests performed is estimated to be 42.5 tests per 100,000 per year with a waiting time of 10 months, while in the USA and Canada the estimations are 427/100,000/year and 370/100,000/year with variable waiting times ranging between few weeks and more than a year depending on the specific district [37].

The reduction in waiting time observed in our study with the transition to ambulatory testing is quiet obvious and could be easily expected. Instead of 2–8 beds availability in the lab (depending on the lab we worked with), we had 20 ambulatory devices available, and the patients got the ambulatory devices immediately after leaving the physician room. Scheduling a PSG sleep study was limited due to beds availability and scheduling procedures. Also patients preferred not to do the test because of difficulties to travel again to the lab from distance and the expenses that such a travel brings. Interestingly the transition to home testing resulted in a fourfold reduction in waiting times for in-lab PSG during 2010-2011 compared to that during 2007-2008, which can probably be explained by the reduced volume of sleep tests performed in the lab, while big part of sleep tests was done at home. Thus, patients with serious comorbidities or unstable conditions, who were not candidates for ambulatory sleep tests, had a benefit from shorter waiting times to in-lab sleep tests.

While the number of sleep tests performed almost doubled itself in 2010-2011 as compared to 2007-2008, there was only 30% increase in the amount of CPAPs purchased during these years. One explanation for this disproportional increase can be that larger part of sleep tests has been done for nonobstructive sleep disorders in 2010-2011 than in 2007-2008 due to the increased availability of the test. Although the home sleep testing was prescribed only for suspected OSA, it may have freed lab space for different diagnoses such as parasomnias or neurological disorders. Another potential explanation is that more patients with relatively low pretest probability were now studied, while previously studies were reserved for patients with higher pretest probability. We do not have data to test this option but we plan to do it in the future. A third potential explanation is that CPAP adjustment procedure in the sleep clinic was improved, in such a way that only compliant patients were directed to purchase the device. While previously free habituation to CPAP was 1 week, followed by a decision by the patient whether to purchase or not, in recent years free habituation time was increased providing the patients a better perspective and a more based decision. Further studies will have to assess whether indeed compliance with CPAP was increased between these years. Finally, another possible explanation is that the home sleep testing underestimated the severity of OSA, resulting in lower CPAP purchase rate. We believe that this is an unlikely explanation. Recently a large scale meta-analysis indicated that the WP100 CHS used is considered an accurate device [38].

Patients' satisfaction was similarly high in both arms, both for patients who underwent in-lab sleep tests and for patients who underwent home sleep tests. For the in-lab studies, patients commended the warm treatment and the close observation they received from the laboratory staff during the night, while patients who underwent home studies indicated the advantage of being studied in their own and familiar environment. Our prestudy hypothesis that satisfaction with home studies will increase was not corroborated. It appeared that patients' satisfaction is the same in the home and in the lab. While convenience is higher in the home, the worry from an unsuccessful test due to its' unattended nature balances the satisfaction score by the patients.

Nevertheless, in a retrospective vision, 56% of patients form the lab arm and 72% of patients from the home arm preferred the home sleep test. We can conclude that despite the close observation the patients get in the lab, the transition to home sleep tests did not impair patients' satisfaction and possibly resulted in an increasing trend to be studied in home. Since the number of participants in our telephone questionnaire was very small, these conclusions should be taken just as preliminary and not necessarily representative. Obviously for better perspective of patients' satisfaction, a substantially larger sample size should be interviewed.

Home test cost is significantly lower than in-lab sleep polysomnography. This explains an over 20% decline in the total cost of sleep tests during 2010-2011 compared to that during 2007-2008, despite the 90% increase in the number of sleep tests done. Recently three large studies were carried out and examined the effect of transition to home sleep tests on the diagnosis and treatment outcome of OSAS. In 2008, Berry et al. randomly divided 106 patients with high risk for OSAS into two arms. One arm was managed by at home sleep tests using Watch-PAT 100 and CPAP autotitration, while the second arm was managed in the laboratory for both diagnosis and CPAP adjustment [25]. In both arms there were similar outcomes in terms of compliance to CPAP treatment, daily CPAP usage, improvement in daytime sleepiness and quality of life, and patients' satisfaction. More recently, two additional studies were published which similarly compared the two arms of diagnosis and CPAP adjustments and concluded that home management was not inferior to in-lab management of OSAS patients [24, 26]. Thus, with similar outcomes between the ambulatory and lab arms, the report in which costs and waiting times are reduced is substantial [26].

Our study has several limitations. First, it was a retrospective data-based study. Thus, the information and data we have regarding the individual patients are very limited. On the other hand, it allows us to report a real field results without bias in patients' behavior or CPAP usage which may occur when a prospective study is performed [39]. In addition, it increases the number of cases being assessed. Second, we compared 2 different periods, assuming the single difference between them is the method of sleep testing. Obviously other changes have occurred during these periods that may have affected the results of this study. One such change is a potential policy alteration of CHS, manifested as different approach of the gatekeeper. Since the economic burden of the HMO did not change, no gatekeeping changes were done in waiting periods to OSA or any other ambulatory tests. However, we believe that potentially other changes are random and not specific for lab or home studies and therefore their effect may not be directional. Finally, we do not have outcome data such as compliance with CPAP or other treatments. However, this was not the focus of our study. We primarily aimed at testing the effect of the home sleep testing on the volume of studies, waiting time, satisfaction, and cost, which we indeed retrieved. Relevant outcome data was studied by others and probably will also be studied in future research.

In conclusion, despite these limitations, we believe that our study, which was a true “field study,” indicates that the transition to unattended home sleep testing in Haifa and West Galilee district of Clalit Health Services improved tests accessibility, reduced waiting times and total costs, and did not impair patients' satisfaction. We also hope that this intervention also contribute to the health of these patients.

## Figures and Tables

**Table 1:** Summary of the patients' satisfaction questionnaires.

Question	Home	Lab-PSG	*P* value
From scale 0 to 10, how uncomfortable was to sleep while being connected to the device? (the higher the result is, the more uncomfortable it is)	2.7	4.1	0.11
How much do you think the study result is true? (0–10)	6.9	7.1	0.84
How much does the diagnostic process influence your decision for treatment? (0–10)	8.9	8	0.14
Estimate your satisfaction from the diagnostic process (0–10)	7.5	8.6	0.2
Do you prefer in-lab or home study? (percent of patients who prefer home study)	72%	56%	0.05*

*P < 0.05.
